# An Electrochemical Sensor Based on Gold Nanodendrite/Surfactant Modified Electrode for Bisphenol A Detection

**DOI:** 10.1155/2020/6693595

**Published:** 2020-12-29

**Authors:** Nguyen Thi Lien, Le Quoc Hung, Nguyen Tien Hoang, Vu Thi Thu, Dau Thi Ngoc Nga, Pham Thi Hai Yen, Pham Hong Phong, Vu Thi Thu Ha

**Affiliations:** ^1^Department of Chemistry, Hanoi University of Science, 19 Le Thanh Tong, Hanoi, Vietnam; ^2^Institute of Chemistry, Vietnam Academy of Science and Technology, 18 Hoang Quoc Viet, Cau Giay, Hanoi, Vietnam; ^3^University of Science and Technology of Hanoi, Vietnam Academy of Science and Technology, 18 Hoang Quoc Viet, Cau Giay, Hanoi, Vietnam

## Abstract

In the present work, we reported the simple way to fabricate an electrochemical sensing platform to detect Bisphenol A (BPA) using galvanostatic deposition of Au on a glassy carbon electrode covered by cetyltrimethylammonium bromide (CTAB). This material (CTAB) enhances the sensitivity of electrochemical sensors with respect to the detection of BPA. The electrochemical response of the modified GCE to BPA was investigated by cyclic voltammetry and differential pulse voltammetry. The results displayed a low detection limit (22 nm) and a linear range from 0.025 to 10 *µ*m along side with high reproducibility (RSD = 4.9% for seven independent sensors). Importantly, the prepared sensors were selective enough against interferences with other pollutants in the same electrochemical window. Notably, the presented sensors have already proven their ability in detecting BPA in real plastic water drinking bottle samples with high accuracy (recovery range = 96.60%–102.82%) and it is in good agreement with fluorescence measurements.

## 1. Introduction

Bisphenol A (BPA) is known as a typical endocrine disruptor in the environment, which is widely used in the production of plastic products [1]. As a result, BPA is found in food and drinking water from product packaging, and therefore humans may routinely ingest amounts of BPA even in trace levels [2]. So far, different analytical methods have been developed and used for the determination of BPA, such as gas chromatography [3], high performance liquid chromatography [4], fluorimetry [5], molecular imprinting [6], and enzyme-linked immunosorbent assay [7]. Electrochemical detection techniques have advantages in relation to these techniques of rapidity, low cost, high sensitivity, simple operation, good selectivity, and real-time detection with *in situ* analysis [8]. Recently, electrochemical techniques have been popularly developed for BPA detection [9–28], especially many publications focus on electrode modification in order to enhance signals of target analytes. Recently, lots of different modifiers have been developed for electrochemical sensors such as carbon black/f-MWCNT composite modified glassy carbon electrode (GCE) which was used for detection of BPA with a detection limit of 0.08 *μ*mol L^−1^ [9], molybdenum disulfide nanoflower-chitosan-Au nanoparticle composites [10], poly(amidoamine)–AuNP-silk fibroin [29], carbon nanotubes-*ß*-cyclodextrin [30], and carboxylic group functionalized CNT-poly(3, 4-ethylenedioxythiophene) [31]. Besides that, gold nanoparticles (AuNP) combined with other materials were of interest to many researchers, such as multiwall carbon nanotubes (MWCNT) plus AuNP modified GCE [11, 32, 33] because Au is the material with extremely high conductivity. As Najib Ben Messaoud et al. [11] showed, a modified electrode was prepared by two steps: drop-casting a desired amount of MWCNT on polished GCE followed by deposition of AuNP on the surface of the previously prepared MWCNT/GCE. With this sensor, the detection limit of BPA reached 4 nM. In this work, an electrochemical sensor for BPA detection consisting of gold nanodendrites (AuNDs) deposited on GCE, which was precovered by cetyltrimethylammonium bromide (CTAB). CTAB is one of the known cationic surfactant which has been extensively employed to enhance the sensitivity of electrochemical sensors [34, 35]. It is also presented that CTAB has a hydrophilic head on one side and a long hydrophobic tail on the other side of its molecule. Also, as reported, surfactants adsorbed at the electrode surface may alter the electrode behavior and accelerate the electron transfer process between the analyte and the electrode surface [36]. In this context, the GCE-supported Au nanodendrites and CTAB (denoted as AuNDs/CTAB/GCE) were demonstrated as an electrochemical sensor, which has sensitive electrochemically active surface areas due to the hierarchical nanodendrites structure and the formation of nanodendrite on the CTAB/GCE was accomplished in just 5 minutes by simple one-step electrodeposition. The electrochemical behavior of BPA on this sensor was studied by cyclic voltammetry (CV) and electrochemical impedance spectroscopy (EIS), and the analytical measurements were performed by differential pulse voltammetry (DPV). The AuNDs/CTAB/GCE sensor has been employed to detect BPA in aqueous samples and important factors related to electrochemical response, such as pH (from 5.0 to 9.0), were examined as well to get the optimal conditions for BPA detection.

## 2. Materials and Methods

### 2.1. Materials and Sensor Preparation

Bisphenol A (BPA) was purchased from Sigma-Aldrich (Structures in Figure S1). GCE (BAS, diameter = 3 mm) was used as a current collector. Phosphate buffer solution (PBS) was prepared by mixing 0.2M KH_2_PO_4_ and 0.2 MK_2_HPO_4_ until the desired pH (in the range from 5.0 to 9.0) was obtained. Potassium ferrocyanide trihydrate (K_4_[Fe(CN)_6_].3H_2_O), potassium ferricyanide (K_3_[Fe(CN)_6_]), and all reagents needed for the construction of Au nanodendrites, HAuCl_4_ (99.999%), KI, H_2_SO_4_, and NH_4_Cl were bought from Sigma-Aldrich. All chemicals were used as received without any further treatments.

The AuNDs/CTAB/GCE sensor was prepared as reported in our previous publication [37, 38]. In brief, 5 *µ*L CTAB was drop-casted on GCE, dried in the atmosphere in about 2 h 30 to 3 h. The dried CTAB/GCE was put in a solution containing 20 mM HAuCl_4_, 1 mM KI, 5 M NH_4_Cl, and 0.5 M H_2_SO_4_, and previous regime published by author's group was followed [37, 38]. A current of -20 mA was applied to it for the preparation of Au nanodendrites by electrodeposition for 30 s. AuNDs/CTAB/GCE was rinsed through by a mixture of EtOH and H_2_O (1 : 3) and kept in a desiccator overnight. A scanning electron microscope (SEM) image of the built Au nanodendrite was obtained (S-4800, Hitachi, Japan) with an electron beam accelerated by a voltage of 15–20 kV to characterize the structure. Fluorescence spectra were obtained with a FluoroMax-4 spectrofluorometer equipped with an Ozone-free xenon arc lamp (150 W), Czerny-Turner monochromators (HORIBA–Japan). All measurements were performed in a 10 mm quartz cell at room temperature. The emission spectra are recorded between 470 and 540 nm with the excitation wavelength at 460 nm.

### 2.2. Electrochemical Measurements of Samples

All of the electrochemical measurements were conducted at room temperature (25°C ± 1) using a three-electrode system, in which an Ag/AgCl electrode and a Pt wire were used as the reference and auxiliary electrode, respectively. A custom-made, multifunctional potentiostat-galvanostat manufactured by this research group (Vietnam Academy of Science and Technology, Hanoi, Vietnam) was used. It was equipped with 12-byte analog-digital and digital-analog converters with two operational amplifiers, and it provided the current resolution down to 0.008 nA.

DPV was used for BPA measurement with a differential step of 0.005 V, a sampling time of 0.04 s, a pulse width of 0.08 s, and a pulse amplitude of 0.05 V. The accumulation conditions of BPA onto AuNDs/CTAB/GCE were conducted at *i* = 0 (under open-circuit potential) with a duration time of 240 s (selected from previous publications [39] and voltammograms were recorded over a range of 0.25 to 0.85 V versus Ag/AgCl. A PBS solution (pH 7) was used as a supporting electrolyte in all measurements.

## 3. Results and Discussion

### 3.1. Surface Morphology of Prepared Sensors

**Figure 1:**
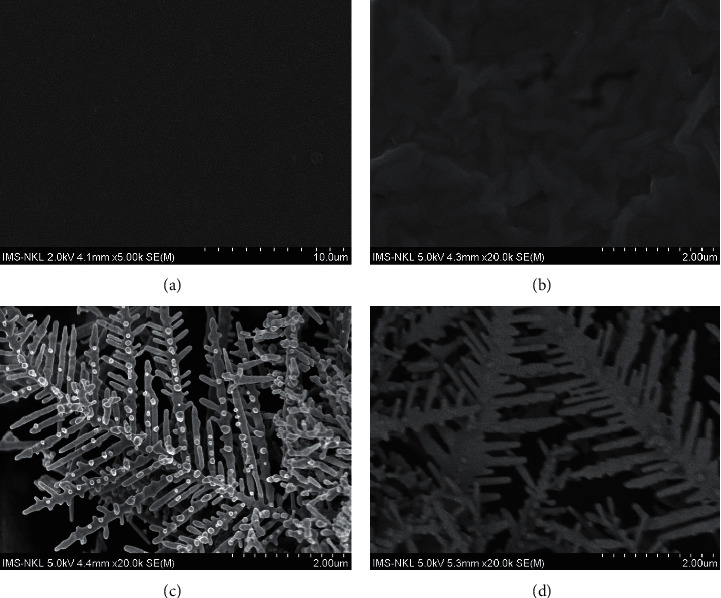
SEM images of (a) GCE, (b) CTAB/GCE, (c) AuNDs/GCE, and (d) AuNDs/CTAB/GCE samples.

The surface morphology of AuNDs/CTAB/GCE was examined using the SEM technique (Figure 1). Au nanodendrites were quickly formed on the electrode surface after 5-minutes of electrodeposition. When CTAB surfactant was not used, the aggregation of gold nanostructures (Figure S2) and the formation of large gold branches (Figure 1(c)) were observed. The use of CTAB has helped to limit those unwanted defects, thus significantly increased the active surface area and provided more access sites for analytes at not only the tip of gold brands but also inside gold branches.

### 3.2. Electrochemical Performance of AuND/CTAB/GCE

**Figure 2:**
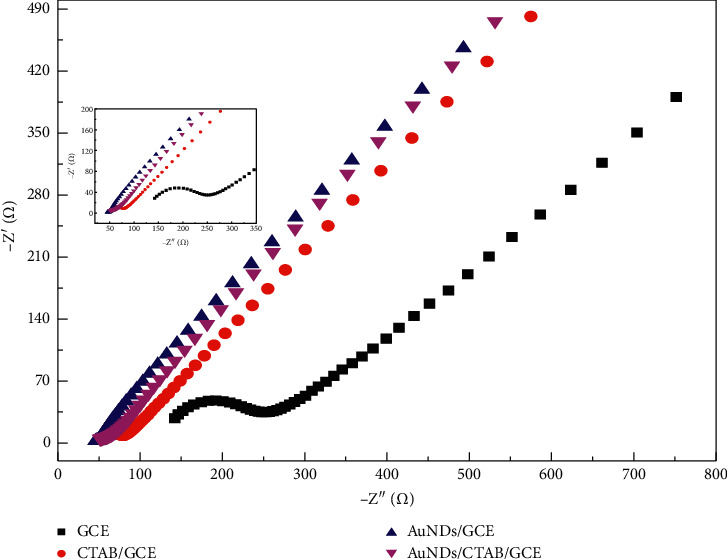
Nyquist diagrams of GCE, CTAB/GCE, AuNDs/GCE, and AuNDs/CTAB/GCE in the solution containing 5 mM Fe(CN)_6_^3−/4−^ and 0.1 M PBS. Parameters are as follows: Frequency range from 0.1 Hz to 10000 Hz, initiative potential: 0.23 V, amplitude: 10 mV, and quiet time of 5 s.

**Figure 3:**
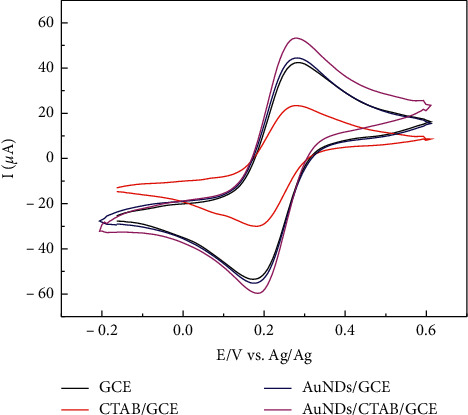
CVs of GCE, CTAB/GCE, AuNDs/GCE, and AuNDs/CTAB/GCE in 5 mM Fe(CN)_6_^3−^ and 0.2 M PBS, from − 0.2 to 0.6 V versus Ag/AgCl.

Before using AuNDs/CTAB/GCE prepared for detection of BPA on GCE, CTAB/GCE, AuNDs/GCE, and AuNDs/CTAB/GCE, electrochemical impedance spectroscopy (EIS) was recorded in 0.2 M PBS containing 5 mM Fe(CN)_6_^3−/4−^. In the Nyquist diagram (Figure 2), the bare GCE exhibited a relatively low electron transfer resistance (*R*_ct_ = 146.3 Ω) with a small semicircle (refer to in inset for further detail). After the drop-casting CTAB and deposition of AuNDs, the electron transfer rate dramatically increased, which resulted from a decrease of the charge transfer resistance (*R*_*ct*_ = 25.4 O only) due to the high conductivity of AuNDs on the electrode surface. Also, CTAB molecules must have aided to improve the electron transfer process of Fe(CN)_6_^3−/4−^ probe at electrode surface [35]. All the results further demonstrate that the electrode is well done with the presence of AuNDs layers on its surface. This result is in good agreement with CV tests (Figure 3).

In Figure 3, it is seen that in a well-defined reversible redox system with a peak separation (ΔE_p_) of ∼80 mV for all cases, the CTAB/GCE exhibits a pretty low electron transfer rate. After deposition of AuNDs, the oxidation peak of this redox probe increased almost three times higher than CTAB/GCE only in terms of electrical conductivity (Figure 3). Also, as stated in [36], CTAB can help in accelerating the electron transfer process between the analyte and the electrode surface. Therefore the total peak current received on AuNDs/CTAB/GCE was the highest.

### 3.3. Effects of Scan Rate (*ν*)

**Figure 4:**
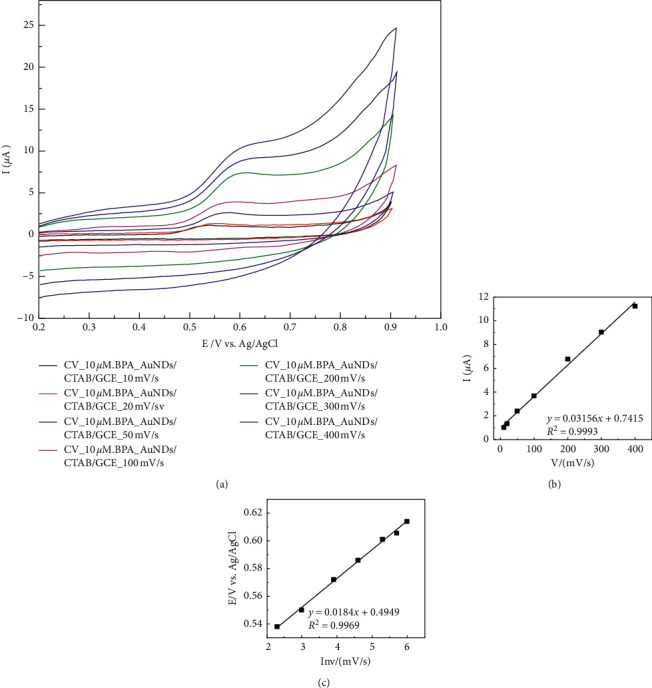
(a) CVs of BPA 10 *µ*m in PBS pH 7 recorded on AuNDs/CTAB/GCE at different scan rates (*ν*): 10, 20, 50, 100, and 200 mVs^−1^. (Inset) Relationship (b) between anodic peak currents and scan rate and (c) between potentials and ln(*ν*).

Kinetics of electrochemical oxidation of BPA on AuNDs/CTAB/GCE was investigated in PBS at pH 7 containing 10 *µ*m target analyte with different scan rates from 10, 20, 50, 100, and 200 mV s^−1^ (Figure 4). Oxidation peak currents appeared at 0.55 V and increased linearly with the increasing scan rate as follows: *i*_*p*,*a*_ = 0.0316 × *ν* + 0.7415 (*R*^2^ = 0.9993), which suggests an adsorption controlled kinetic process on the modified electrode surface AuNDs/CTAB/GCE [19–28]. As seen in Figure 4, the oxidation peak potential (*E*_*p*,*a*_) slightly shifted to more positive potentials with the increase of the scan rate. The dependence between *E*_*p*,*a*_ and the ln(*v)* was given: *E*_*p*_,_*a*_(V) = 0.0184 × ln(*v)* + 0.4949 (*R*^2^ = 0.9969) (inset of Figure 4(b)), and the number of electrons transferred estimated from Laviron quation is as follows [40]:

(1) $$\begin{array}{c} E_{p,a}=\;E^{o}+\left(\frac{\text{RT}}{\alpha nF}\right)\text{ln}\left(\frac{\text{RT}k^{o}}{\alpha nF\;}\right)+\left(\frac{\text{RT}}{\alpha nF}\right)\text{ln}\left(v\right), \end{array}$$

where *v* is the scan rate, *n* is the number of electrons transferred, *α* is the electron transfer coefficient, k^o^ is the standard rate constant of the reaction, and *R*, F, and *T* are gas constant, Faraday constant, and absolute temperature, respectively. As the slope of the plot of *E*_*p*,*a*_ versus ln(*v*) (equal to RT/*α*_n_F) is 0.0184, the value of *α*_*n*_ was about 1.3. Also, from Laviron [40], *α* for an irreversible electrode process is assumed to be 0.5. Therefore, the number of electrons transferred (*n*) for electrooxidation of BPA is around 2 [26–28].

### 3.4. Effect of pH

**Figure 5:**
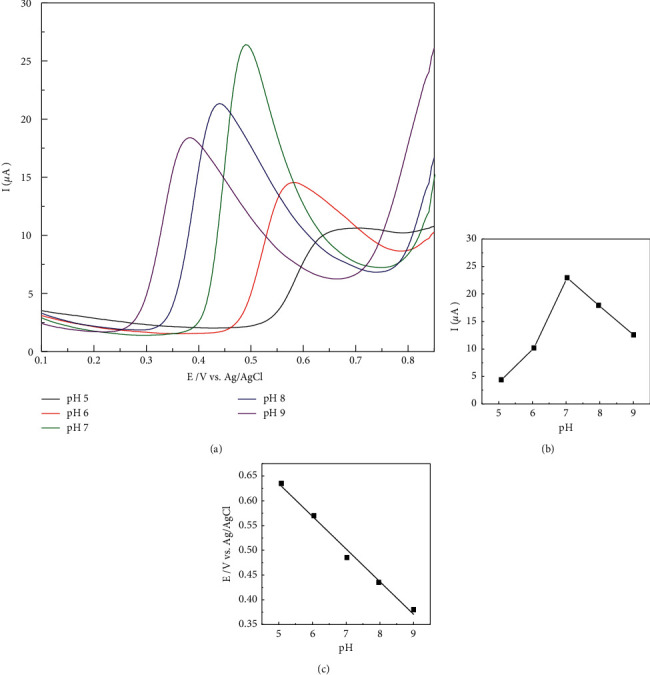
DPVs of 50 *µ*m BPA on AuNDs/CTAB/GCE in PBS pH 7 with different pH: 5.0; 6.0; 7.0; 8.0, and 9.0 (a). Currents varied by pH (b) and relationship between potentials and pH (c).

It is known that the electrochemical oxidation of BPA followed a proton coupled electron transfer mechanism, i.e., BPA ⇌ oxidized product + ne^–^ + nH^+^ (*n* = 1 or 2); the reaction rate is directly depending on the electron flux and the concentration of proton in solution. Consequently, the pH becomes one of the key parameters impacting the performance of the presented sensors. DPVs of BPA (*C* = 50 *µ*m) were recorded by sweeping the potential from 0.1 V–0.9 V in PBS buffer with pH ranging from 5.0–9.0 at interval 1.0. It was found that the peak potential is shifted to a more negative direction at higher pH values (Figure 5(a)).

By plotting the variation of peak potential depending on pH values, a linear relationship is obtained, i.e., *E*_*p*, *a*_ = −0.0659 pH + 0.9642, *R*^2^ = 0.9869. A slope of 0.065 V/pH was pretty close to the theoretical value of 0.059 V/pH, showing that the transfer of electrons was accompanied by an equal number of protons in electrode reaction. According to the calculation in [41] −0.065*x*/*n* = −0.059, where *n* is the transferred electron number and *x* is the number of protons involved in the reaction, as mentioned above, the number of electrons transferred is about 2, the electrochemical oxidation of BPA is a two-electron and two-proton process, which is in good agreement with previous publications [26–28] as below.


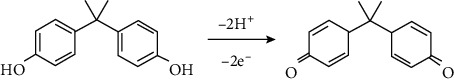


### 3.5. Detection of BPA Using AuNDs/CTAB/GCE

**Figure 6:**
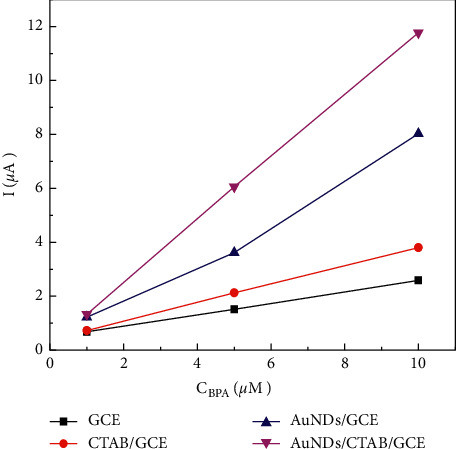
Peak current of BPA at 1.0, 5.0, and 10.0 *µ*m on GCE, CTAB/GCE, AuNDs/GCE, and AuNDs/CTAB/GCE recorded in PBS pH 7.

In order to compare electrochemical signals BPA on four materials used for the sensor, GCE, CTAB/GCE, AuNDs/GCE, and AuNDs/CTAB/GCE, DPV signals were recorded in PBS pH 7.  Figure 6 describes peak currents of BPA on four above materials at 1.0, 5.0, and 10 *µ*m. It is seen that currents measured on AuNDs/CTAB/GCE are always highest and it is almost 10-, 4-, and 2-fold compared with it on GCE, CTAB/GCE, AuNDs/GCE, respectively. Also, it was observed visually that with AuNDs/GCE only, AuNDs layers easily sloughed from the GCE surface. By measuring CVs of AuNDs/CTAB/GCE at different scan rates in 0.2 M PBS containing 5 mM Fe(CN)_6_^3−/4−^, electrochemical active surface of this sensor is not a significant difference in comparison with bare GCE (Figure S3). Therefore, it is assumed that the increase of peak current was due to the fact that AuNDs might provide more electroactive sites for BPA electrooxidation, while CTAB could improve adherence of targeted molecules and promote electron transfer process at electrode surface [35].

### 3.6. Calibration Curve

**Figure 7:**
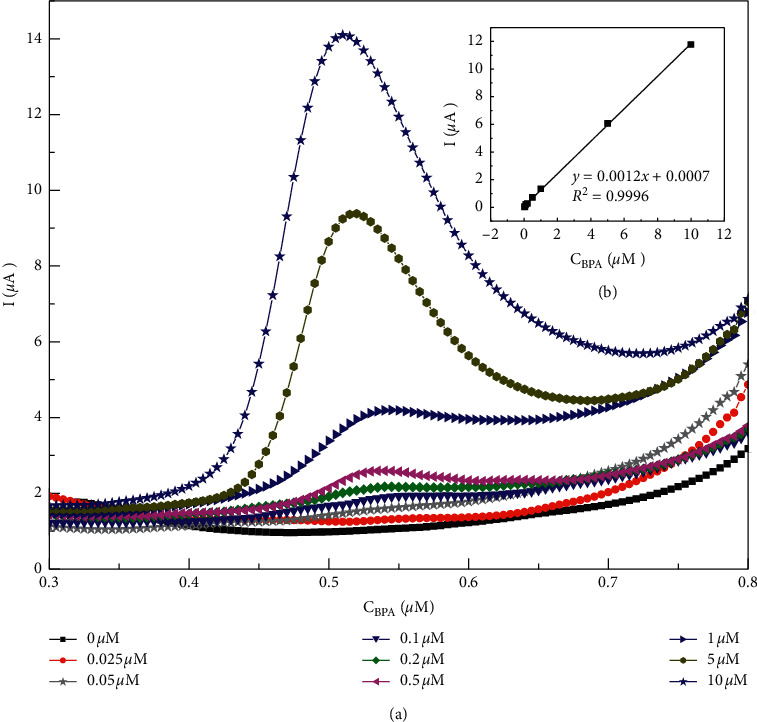
(a) Voltammograms recorded from BPA in the concentration range from 0.025 to 10 *µ*m using AuNDs/CTAB/GCE and (b) the variations of peak intensities according to the concentration change.

Figure 7(a) shows voltammograms of all the BPA samples measured using selected AuNDs/CTAB/GCE under optimized conditions. BPA peak heights increased with an increase in concentrations with the regression equation as follows: *i*(*µ*A) = 0.0012 × *C*_BPA_(*µ*m) + 0.007 (*R*^2^ = 0.9996) (Figure 7(b)).

The limit of detection of the sensor (LOD) was estimated (LOD=3.3  × *S* *D*/*b*; *SD*: standard deviation of ordinate intercept, *b*: slope of regression line) to be 22 nm for BPA in PBS (pH 7). It showed that the LOD of AuNDs/CTAB/GCE sensor was comparable with those of other electrode-based sensors (Table 1) and much lower than the allowable level of BPA declared by WHO [42]. The excellent performance of AuNDs/CTAB/GCE based sensor for BPA benefits from the high conductivity, large area of AuNDs, and charge transfer promotion along long alkane chain of CTAB [35].

### 3.7. Reproducibility, Repeatability, and Interference Study

The reproducibility was tested using seven separated sensors (Figure S4). The magnitudes of error bars are quite small and the average relative standard deviations (RSDs) in the measurements of BPA were less than 5.0%, thereby confirming the reproducible formation of AuNDs/CTAB on GCE. Repeatability tests have been done with 5 consecutive measurements using one AuNDs/CTAB/GCE sensor. RSDs received for 10 *µ*m BPA in solutions was 4.37%, less than 5.0% as above.

Finally, the selectivity of as-prepared sensors was evaluated in the presence of Cu^2+^/Pb^2+^/Cd^2+^ and 4-nitrophenol as interferants. The concentrations of interferants were designedly fixed at 100 *µ*m, which is 20 times greater than BPA concentration. No significant changes in current intensity observed, suggesting no interference effect due to other compounds in this study (Figure S5).

### 3.8. Real Sample Analysis

To open the application, AuNDs/CTAB/GCE was further used to determine BPA quantities in real samples prepared from a plastic drinking water bottle. Sample preparation from plastic pieces was treated as in [43]. Briefly, the plastic sample of LaVie bottles, a popular commercial product in Vietnam, were cut into small pieces and washed several times with double distilled water. After vacuum drying, 1.0 g of the plastic sample and 50 mL of purified water were added to the flask, sealed, and heated at 70°C for 48 hours. Finally, the mixture is filtered by a 45 *µ*m filter. The as-prepared solution was divided into two parts: one for fluorescence measurement and the second part for electrochemical measurement in PBS pH 7. DPV voltammograms are shown in Figure S6. The emission spectra of BPA appeared at a wavelength of 506 nm (Figure S6, (b)).  Table 2 shows concentrations of BPA spiked in the treated solution using prepared AuNDs/CTAB/GCE and the calculated recoveries by both techniques.

The concentration determinations using AuNDs/CTAB/GCE were accurate, with a recovery range of 96.60%–102.82%. These were in agreement with data obtained using a FluoroMax-4 spectrofluorometer. Therefore, the detection performance of AuNDs/CTAB/GCE was acceptable for this purpose.

## 4. Conclusion

In this paper, the GCE-supported Au nanodendrite and CTAB (denoted as AuNDs/CTAB/GCE) have been developed by simple one-step electrodeposition. The AuNDs/CTAB/GCE sensor was able to detect BPA in a large concentration range (0.025–10 *µ*m) with a relative detection limit of 22 nm. Sensor-to-sensor reproducibility was estimated by measuring seven separate prepared AuNDs/CTAB/GCE sensors and the obtained RSDs were only less than 5.0%. Real samples extracted from plastic drinking bottles were also tested and the determined concentrations were in good recoveries (96.60%–102.82%) and comparable with other conventional methods. Furthermore, AuNDs/CTAB/GCE should be employable as an electrochemical sensor for on-site water analysis with low cost and without the serious worry of environmental contamination.

## Figures and Tables

**Table 1:** Comparison of sensing performances of electrochemical sensors for detection of BPA.

Electrode configuration	Method	Linear range (*µ*m)	LOD (*µ*m)	Ref.
CB/f-MWCNTs	CV	0.1–130	0.08	[9]
AuNPs/MoS_2_/GCE	CV	0.05–100	5 × 10^−3^	[10]
SWCNTs/CD/GCE	DPV	0.0108–18.5	1 × 10^−3^	[30]
NPs/MWCNT/GCE		0.01–0.7	4 × 10^−3^	[11]
rGO-NiS	DPV	4.38 × 10^−3^–0.438	1.75 × 10^−3^	[12]
Cu-BTC/GCE	DPV	5 × 10^−3^–2	0.72 × 10^−3^	[13]
Fe_3_O_4_NPs-CB	DPV	0.1 × 10^−3^–50	0.031 × 10^−3^	[14]
RGO-Ag/GCE	DPV	1–80	0.54	[15]
Rh_2_O_3_-GO/GCE	CV	0.6–40	0.12	[16]
Cu-Zn/GO/GCE	SWV	3 × 10^−3^–100 and 350–20000	0.88 × 10^−3^	[17]
GO-CNTs-Fe_3_O_4_	DPV	3 × 10^−3^–0.2 and 0.2–30.0	1 × 10^−3^	[18]
CTAB/MWCNTs/PGE	SWV	2 × 10^−3^–0.808	0.134 × 10^−3^	[19]
CS/N-GS/GCE	CV	0.01–1.3	5 × 10^−3^	[20]
BmimPF_6_/GN/GCE	LSV	0.02–2000	8 × 10^−3^	[21]
GA/MWCNT-NH_2_	DPV	0.1–10	0.02	[22]
Cu_2_O-rGO	DPV	0.1–80	0.053	[23]
AuPdNPs/GNs	DPV	0.05–10	8 × 10^−3^	[24]
PCE/PEDOT/BMIMBr	DPV	0.1–500	0.02	[25]
MoS2-SPAN/GCE	DPV	0.001–1.0	0.6 × 10^−3^	[26]
ELDHs/GCE	DPV	0.02–1.51	6.8 × 10^−3^	[27]
CTAB/Ce-MOF/GCE	DPV	0.005–50	2 × 10^−3^	[28]
AuNDs/CTAB/GCE	DPV	0.025–10	22 nM	This work

AuNPs = gold nanoparticles, CPE = carbon paste electrode, NPs = nanoparticles, ILs : ionic liquids, BTC = benzenetricarboxylic, GO = graphene oxide, ErGO = electrochemical reduced graphene oxide, GCE = glassy carbon electrode; SWV = square wave voltammetry, DPV = differential pulse voltammetry, CV = cyclic voltammetry, LSV = linear sweep voltammetry, MWNT = multiwalled carbon nanotubes, GN = graphene, CTAB = cetyltrimethylammonium bromide, MOF = metal organic frames, PGE = pencil graphite electrode, CS = chitosan, GS = graphene sheet, BmimPF_6_ = -butyl-3-methylimidazoliumhexafluorophosphate.

**Table 2:** Determined BPA concentrations spiked in the real sample using AuNDs/CTAB/GCE and by Fluorescence methods.

Sample	BPA (*µ*m)				
	Added	Found		Recovery (%)	
		By AuNDs/CTAB/GCE sensor	By fluorescence	By AuNDs/CTAB/GCE sensor	By fluorescence
Solution from LaVie plastics bottle	0	Not found	Not found	—	—
	5.00	4.98 ± 0.06	4.88 ± 0.07	99.53	97.23
	10.00	10.28 ± 0.14	10.23 ± 0.09	102.83	102.26
	15.00	14.49 ± 0.29	14.89 ± 0.33	96.60	99.20

The recovery in each case is also shown.

## Data Availability

The graphical abstract, structure of study compound, SEM images at a larger scale, CVs and DPVs of sensor's reproducibility, and real sample analysis data used to support the findings of this study are included within the supplementary information files.

## Supplementary Materials

Supplementary Materials Fig. S1:structure of the studied compound. Fig. S2: SEM images of AuNDs created on GCE without (a) and with CTAB layer (b). Fig. S3: CVs of AuNDs/CTAB/GCE in 5 mM K_3_Fe(CN)_6_/K_4_Fe(CN)_6_ + 0.2 M PBS at different scan rates and used for calculation of electroactive surface area (ECSA) Fig. S4: reproducibility of seven AuNDs/CTAB/GCE sensors at 10 *µ*m BPA in PBS pH 7. Fig. S5: voltammograms of BPA 5 *µ*m on AuNDs/CTAB/GCE before and after adding interferents at concentrations 20 times higher than that of analyte BPA with Cd^2+^, Pb^2+^, and Cu^2+^ (a) and with 4-nitrophenol (b). Fig. S6: voltammograms of solution extracted from plastic drinking water bottle spiked BPA at different concentrations recorded on a AuNDs/CTAB/GCE sensor (a) and by the fluorescence method (b).
