# Supplementary material for: An Electrochemical Sensor Based on Gold Nanodendrite/Surfactant Modified Electrode for Bisphenol A Detection
Source: J Anal Methods Chem. 2020 Dec 29;2020:6693595. doi: 10.1155/2020/6693595 (PMC7785347; doi:10.1155/2020/6693595)
Supplement: Supplementary Materials — Fig. S1:structure of the studied compound. Fig. S2: SEM images of AuNDs created on GCE without (a) and with CTAB layer (b). Fig. S3: CVs of AuNDs/CTAB/GCE in 5 mM K3Fe(CN)6/K4Fe(CN)6 + 0.2 M PBS at different scan rates and used for calculation of electroactive surface area (ECSA) Fig. S4: reproducibility of seven AuNDs/CTAB/GCE sensors at 10 µm BPA in PBS pH 7. Fig. S5: voltammograms of BPA 5 µm on AuNDs/CTAB/GCE before and after adding interferents at concentrations 20 times higher than that of analyte BPA with Cd2+, Pb2+, and Cu2+ (a) and with 4-nitrophenol (b). Fig. S6: voltammograms of solution extracted from plastic drinking water bottle spiked BPA at different concentrations recorded on a AuNDs/CTAB/GCE sensor (a) and by the fluorescence method (b). [file 6693595.f1.pdf]

# An Electrochemical Sensor Based on Gold Nanodendrite/Surfactant Modified Electrode for Bisphenol A Detection

Nguyen Thi Lien[1], Le Quoc Hung[2], Nguyen Tien Hoang[3], Vu Thi Thu[3], Dau Thi Ngoc Nga[3], Pham Thi Hai Yen[2], Pham Hong Phong[2] and Vu Thi Thu Ha[2,3,*]

[1]Department of Chemistry, Hanoi University of Science, 19 Le Thanh Tong, Hanoi, Vietnam.

[2]Institute of Chemistry, Vietnam Academy of Science and Technology, 18 Hoang Quoc Viet, Cau Giay, Hanoi, Vietnam.

[3]University of Science and Technology of Hanoi, Vietnam Academy of Science and Technology, 18 Hoang Quoc Viet, Cau Giay, Hanoi, Vietnam.

Corresponding author: havt@ich.vast.vn

*(Supplementary Information)*

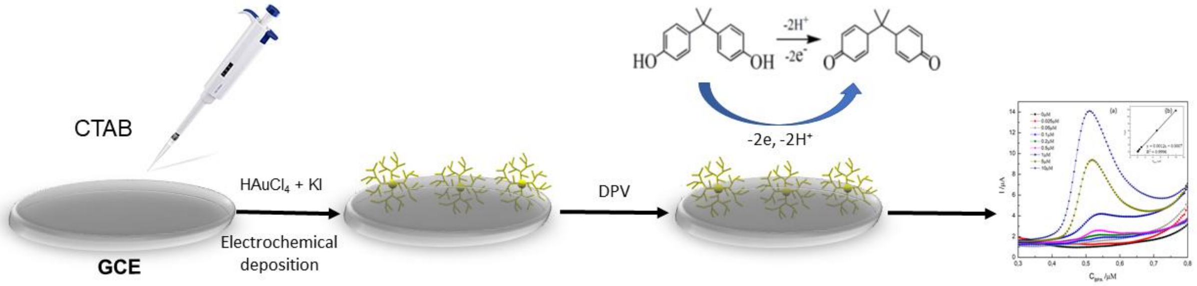


Graphical abstract

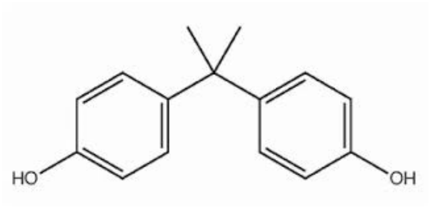


Fig. S1: Structure of studied coumpound

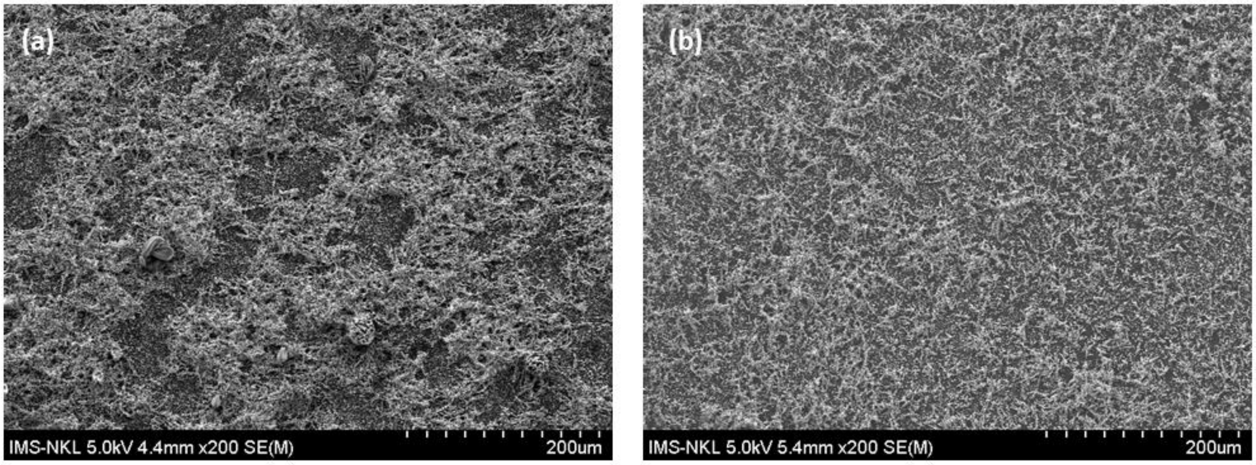


Fig. S2: SEM images of AuNDs created on GCE without (a) and with CTAB layer

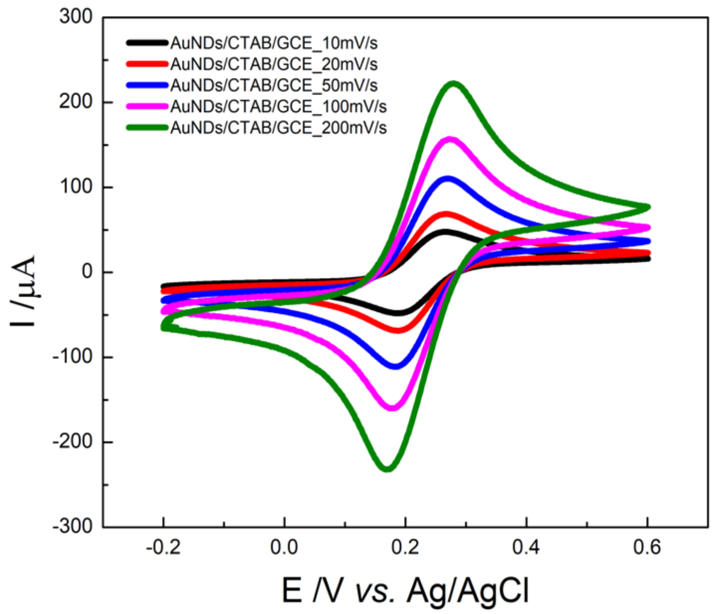


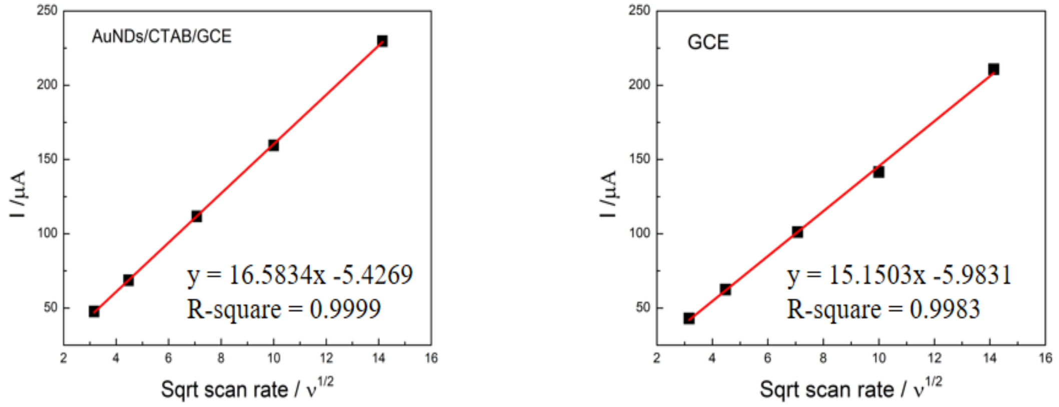


$$Randle - Sevick\ equation:$$

$$I_p = (2.69 \times 10^5) n^{3/2} A C D^{1/2} v^{1/2}$$

$$A\ is\ the\ active\ surface\ area\ (ECSA)(cm^2)$$

$$D\ is\ the\ diffusion\ coefficient\ of\ [K_3Fe(CN)_6]\ (6.605 \times 10^{-6}\ cm^2 s^{-1})$$

$$n = 1\ is\ the\ number\ of\ transferred\ electrons\ for\ [Fe(CN)_6]^{3-/4-}\ redox\ couple$$

$$C\ is\ the\ bulk\ concentration\ of\ [K_3Fe(CN)_6]\ (5\ mM)$$

Fig. S3. CVs of AuNDs/CTAB/GCE in 5 mM $K_3Fe(CN)_6/K_4Fe(CN)_6$ + 0.2 M PBS at different scan rates and used for calculation of electroactive surface area (ECSA)

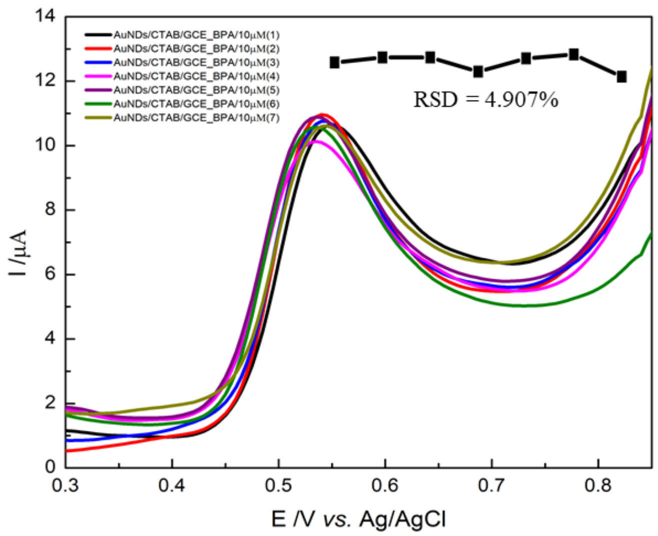


Fig. S4. Reproducibility of seven AuNDs/CTAB/GCE sensors at 10 μM BPA in PBS pH 7

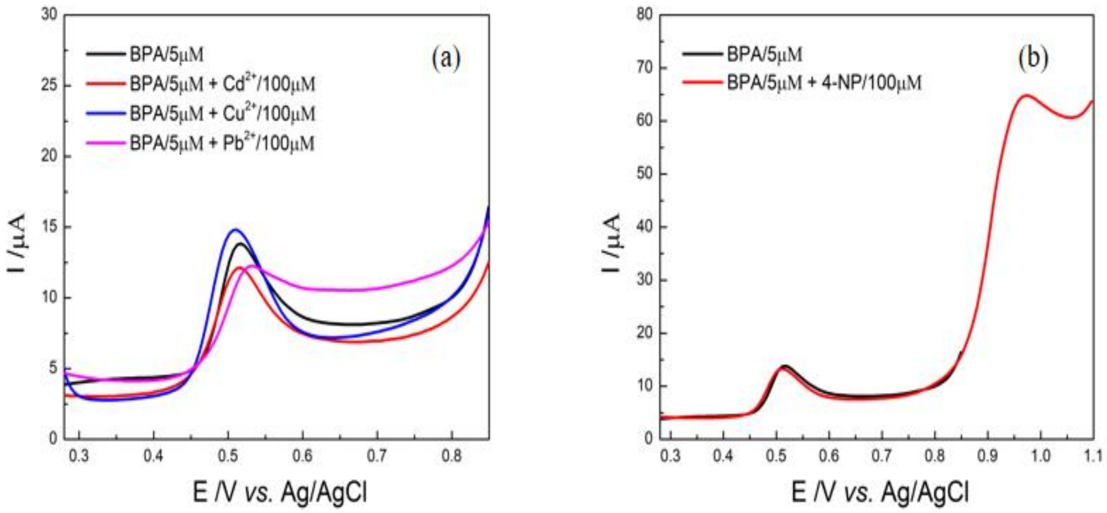


Fig. S5. Voltammograms of BPA 5 μM on AuNDs/CTAB/GCE before and after adding interferents at concentrations 20 time higher than that of analyte, BPA with $Cd^{2+}$, $Pb^{2+}$, $Cu^{2+}$ (a) and with 4-nitrophenol (b)

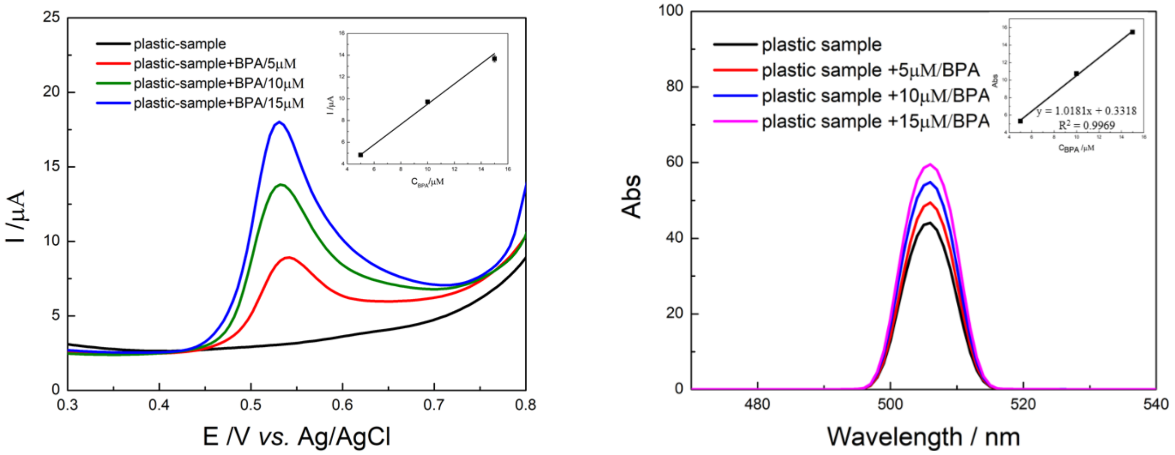


(a) (b)

Fig. S6. Voltammograms of solution extracted from plastic drinking water bottle spiked BPA at different concentrations recorded on AuNDs/CTAB/GCE sensor (a) and by fluorescence method (b)
